# Prevalence of gonococcal and chlamydial infections among men who have sex with men in sub-Saharan Africa: a systematic review and meta-analysis

**DOI:** 10.1186/s13643-024-02704-z

**Published:** 2024-11-16

**Authors:** Kehinde Charles Mofolorunsho, Vinogrin Dorsamy, Chauntelle Bagwandeen, Nathlee Samantha Abbai

**Affiliations:** 1 School of Clinical Medicine Laboratory, College of Health Science, Mandela School of Medicine, University of KwaZulu-Natal, NelsonDurban, South Africa; 2 School of Laboratory Medicine and Medical Sciences, College of Health Sciences, University of KwaZulu-Natal, Durban, South Africa; 3 School of Nursing and Public Health, College of Health Sciences, University of KwaZulu-Natal, Durban, South Africa

**Keywords:** Men who have sex with men, *Neisseria gonorrhoeae*, *Chlamydia trachomatis*, Sub-Saharan Africa

## Abstract

**Background:**

Men who have sex with men (MSM) are disproportionately affected by sexually transmitted infections (STI) including *Neisseria gonorrhoeae* (Ng) and *Chlamydia trachomatis* (Ct). The lack of robust data on STIs among African MSM has limited the development of evidence-based screening strategies. This study aimed at documenting the pooled prevalence of Ng/Ct among MSM in sub-Saharan Africa (SSA).

**Methods:**

This systematic review was performed according to the Preferred Reporting Items for Systematic Review and Meta-analyses (PRISMA) 2020 guidelines. Relevant articles from the following databases were searched: PubMed, Scopus, ISI Web of Science, and the Directory of Open Access Journals (DOAJ). Eligible studies reported on the prevalence of Ng/Ct among the MSM population in SSA. Publication bias was assessed using the Hoy tool, Doi plot, and LFK ratio. Due to heterogeneity among studies, subgroup analyses were performed using the MetaXL add-on tool for Microsoft Excel.

**Results:**

Of 525 articles screened, 20 were selected for inclusion. Six were cross-sectional, four had a prospective cohort study design, and one was an epidemiological study. The pooled prevalence of Ng/Ct in MSM was 27% (95% CI, 19–39%), with an *I*^2^ of 98% signifying heterogeneity among the studies. Subgroup analysis by country revealed South Africa had the highest prevalence (38%).

**Discussion:**

Interpretation

The high prevalence of Ng/Ct infection among MSM in SSA is of concern.

Limitations

Due to limited data available on Ng/Ct prevalence, the true prevalence of SSA and its associated risk factors is uncertain.

**Conclusion:**

As the first study to systematically review the available literature on STI prevalence among the MSM population in SSA, it showed the burden of Ng/Ct is higher than in other regions, warranting the strengthening of health systems to improve education, testing, and treatment in MSM population.

**Systematic review registration:**

PROSPERO CRD42022327095.

**Supplementary Information:**

The online version contains supplementary material available at 10.1186/s13643-024-02704-z.

## Background

Men who have sex with men (MSM) are disproportionately affected by sexually transmitted infections (STIs) including *Neisseria gonorrhoeae* (Ng) and *Chlamydia trachomatis* (Ct) [1–4], accounting for most patients diagnosed with rectal Ng or Ct [5]. Because STIs are often asymptomatic and remain frequently undiagnosed and untreated [6], they continue to pose a significant public health challenge globally. Sexually transmitted infections exert a substantial disease burden [7] and are acquired by more than a million individuals daily, worldwide [8]. An estimated 374 million new infections with one of four curable STIs (gonorrhea, chlamydia, syphilis, and trichomoniasis) are acquired each year [8].

*Neisseria* *gonorrhoeae* and *Chlamydia* *trachomatis* infections are the two most prevalent curable STIs worldwide [9–11]. Approximately 87 million new cases of gonorrhea were reported to have occurred among 15–49-year olds in 2016, with an incidence rate of 26 cases per 1000 men, whereas an estimated 127.2 million new cases of chlamydia occurred globally every year [12]. In Africa, gonorrhea and chlamydia accounted for 11.4 and 12 million new cases per year, respectively [13, 14].

Gonorrhea, which is caused by Ng is highly prevalent in less-developed countries and lower-middle-income countries [12, 15]. The prevalence of which varies by anatomic sites (whether urethral, rectal, or oropharyngeal) [16] and the methods of detection, e.g., Gram’s stain, standard culture, and molecular test [17]. Chlamydia, an equally concerning sexually transmitted infection caused by Ct [18], has been on the rise since 1995, and it is now the most pervasive STI [10, 19, 20], especially among untreated asymptomatic patients [21]. *Chlamydia* *trachomatis* has been implicated in serious complications such as pelvic inflammatory disease (PID), ectopic pregnancy, tubal infertility, and chronic pelvic pain [22, 23] and has also been associated with non-gonococcal urethritis and epididymitis [24].

Men who have sex with men have, in recent years, become the group at greater risk of acquiring STIs worldwide: higher than female sex workers, as well as the general population [25–28]. Extragenital STIs in MSM are frequent [6, 29, 30], mostly asymptomatic [16], and where undetected and untreated, can contribute substantially to further spread [31]. Factors such as increased numbers of sexual partners, increased unprotected anal sex, and increased recreational drug use including chem-sex [32] facilitate the acquisition of STIs among this key population.

According to data from Europe, the USA, and China, MSM have a high burden of HIV and other STIs [33]. In sub-Saharan Africa (SSA), data on bacterial STIs among MSM are sparse [34], because access to this sub-population in many countries of the region remains generally difficult, especially in light of their potential involvement in epidemiological research [35]. This observation is largely due to the criminalization of their sexual orientation [35, 36], stigma, and discrimination by healthcare workers [36, 37]. Social and religious factors also play an important role in limiting research in this sub-population [34].

The lack of robust data on STIs among African MSM [38] has limited the development of evidence-based screening strategies [9]. The major objective of this study was to conduct a systematic review aimed at documenting the pooled prevalence of gonorrhea and chlamydia in MSM in SSA. Other objectives were to describe chlamydial and gonococcal infections diagnosed by syndromic management or laboratory testing and associated risk factors for the prevalent infections in this key population.

## Methods

This systematic review was conducted according to the published protocol [39] registered (CRD42022327095) with the International Prospective Register of Systematic Reviews (PROSPERO). The present review was reported based on the Preferred Reporting Items for Systematic Review and Meta-analyses (PRISMA) 2020 guidelines [40].

### Search strategy

A systematic search of peer-reviewed articles was performed in all the electronic databases listed in the protocol. Search terms were used and their synonyms were identified using the Medical Subject Headings (MeSH). The uniterms and Boolean operators in English used in the search strategies included (Men who have sex with men OR gay) AND ((*Neisseria gonorrhoeae* OR *N. gonorrhoeae* OR Gonorrhoeae infection OR Gonorrhoeae) AND (*Chlamydia trachomatis* OR *C. trachomatis* OR *Chlamydia infection* OR Chlamydia)) AND (Africa OR sub-Saharan Africa OR Western Africa OR Eastern Africa OR Southern Africa OR Central Africa). A combination of relevant key words with names of each of the countries in SSA was also used in the search strategy. Regarding the year of article publication, no restrictions were made to the search.

### Study selection

The selection of eligible studies was based on the criteria listed in the protocol [39]. Original research articles written in the English language that quantified the prevalence of gonorrhea and/or chlamydia, and described data from MSM in SSA, 15 years and older were included. Studies were excluded if they assessed non-human subjects, computed incident infections only, were conducted in countries other than countries in SSA, were published in languages other than English, and were unavailable in full text. Case reports, short reports, letters, notes, conference abstracts, and review articles were also excluded.

### Data extraction and quality assessment

The titles and abstracts of retrieved articles were screened following the removal of duplicates, and full article screening was conducted for their eligibility. Eligible articles were retrieved and exported to the Endnote version 20 reference manager. A hand search of the reference list of all selected articles was also performed to be more comprehensive in the search strategy. Full article screening based on eligibility criteria was then conducted by two reviewers. For the collection of data from eligible studies, a data extraction sheet was designed using Microsoft Excel. This data extraction sheet was piloted and edited through an iterative process [39]. Screening, data extraction, and quality appraisal were independently performed by two reviewers (KCM and VD). Any disagreement in the appraisal of study quality was discussed, and consensus was reached after review by the third and fourth reviewers (NSA and CB). Furthermore, the third and fourth reviewers (NSA and CB) independently verified all extracted data. The study selection process was reported using a PRISMA flowchart [40].

### Risk of *bias* and quality assessment

The risk of bias tool for prevalence studies was used to evaluate the quality and risk of bias of the included studies for the review and meta-analysis. This tool uses a 10-item rating scale to assess the internal and external validity of studies [41] (see Additional file 1: Table S1). Each of the 10 items was rated as either low or high risk of bias, and the overall risk of bias was determined according to the number of high risk of bias per study (low: ≤ 2; moderate: 3–4; and high: ≥ 5) [42]. Insufficient information in a given study related to 10 items was regarded as a high risk of bias [43, 44]. The quality of evidence provided by the included studies was established using the Grading of Recommendations, Assessment, Development and Evaluation (GRADE) [45] tool taking into account the risk of bias, indirectness of evidence, inconsistencies, imprecision, and publication bias. The quality assessment was carried out independently by two reviewers, and disagreements were settled by discussion.

### Data synthesis and analysis

All extracted data was analyzed using the MetaXL add-on for Microsoft ® Excel [46, 47]. Data was presented using tables, and the results were graphically presented in forest plots. Heterogeneity among studies was determined using the *I*^*2*^, with a value over 50% indicative of greater heterogeneity. MetaXL software was used to calculate pooled prevalence and a subgroup analysis by country was conducted to detect possible sources of heterogeneity. The Doi plot and LFK ratio were employed to visualize publication bias and asymmetry, respectively [48]. A sensitivity analysis for the overall pooled prevalence of Ng and Ct was also conducted to assess the impact of individual studies on the pooled prevalence.

## Results

### Characteristics of studies

**Fig. 1:**
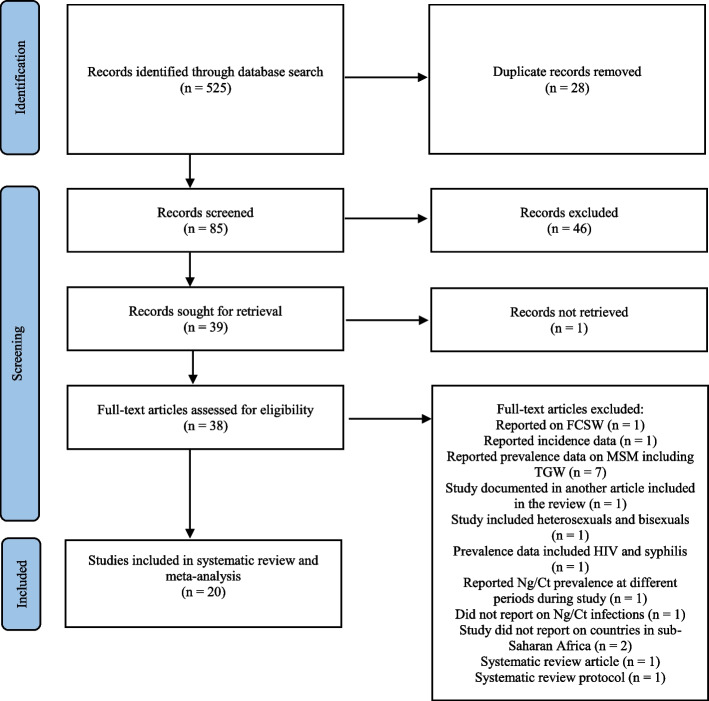
PRISMA flow diagram describing selection of studies for systematic review of gonorrhea and chlamydia prevalence among MSM in SSA [40]

The initial search retrieved 525 potential articles, of which 28 were duplicates. A total of 38 articles were found eligible for full-text screening, of which 18 articles were excluded due to reported on female commercial sex workers (FCSW) (*n* = 1), reported incidence data (*n* = 1), reported prevalence data on MSM including transgender women (TGW) (*n* = 7), study documented in another article included in the review (*n* = 1), study included heterosexuals and bisexuals (*n* = 1), reported prevalence data on STIs including HIV and syphilis (*n* = 1), reported Ng/Ct prevalence at different periods during the study (*n* = 1), did not report on Ct/Ng infections (*n* = 1), study did not report on countries in SSA (*n* = 2), systematic review article (*n* = 1), and systematic review protocol (*n* = 1) (Fig. 1). Twenty articles that reported on the prevalence of gonorrhea and chlamydia infections in MSM in SSA were identified as fulfilling the criteria for inclusion in the analysis (Table 1) [34, 38, 49–66]. All the studies were conducted in the major cities of the countries including Togo [59], Kenya [50, 53, 58, 62, 64, 66], South Africa [34, 38, 54, 61, 65], Nigeria [56, 57], Tanzania [52], Uganda [55], Côte d’Ivoire [51], and Senegal [49]. Two studies were conducted across four countries (Burkina Faso, Mali, Togo, and Côte d’Ivoire) [60, 63]. The lowest prevalence rates for Ng and Ct were reported from Uganda [55]. The highest prevalence rates for Ng and Ct were reported from Kenya [58, 66]. The included studies ranged in publication year from 2005 to 2023. The total number of participants pooled for this study was 5818, and the sample size for each included study ranged from 43 to 698 (Table 1). For the purpose of our study, we selected MSM based on description and selection criteria distinguished by the included studies. For example, MSM was defined as men who were born male, assigned male sex at birth, self-declared to have sex with other men, engaged in consensual receptive/insertive anal and/or oral sexual intercourse with another man, and self-reported as MSM.

**Table 1:** Characteristics of studies included in meta-analysis

Author	Country	Region	Study design	Sampling	Number of MSM tested	Diagnostic method used	Specimen	Infection	Prevalence (%)
Quilter 2018 [58]	Kenya	Kisumu	Not reported	Snowball	698	NAAT	Urine	Ng	47.2
						Microscopy	Rectal swab	Ct	25.0
Rebe 2015 [54]	South Africa	Cape Town	Cross-sectional	RDS	200	NAAT	Urine	Ng	16.0
							Rectal swab	Ct	12.0
							Pharyngeal swab		
Ramadhani 2016 [56]	Nigeria	Lagos	Prospective cohort	RDS	492	NAAT	Urine	Ng/Ct	11.4
		Abuja					Rectal swab		
Crowell 2018 [57]	Nigeria	Lagos	Cohort	RDS	420	NAAT	Urine	Ct	15.7
							Rectal swab		
Ross 2014 [52]	Tanzania	Dar es Salaam	Cross-sectional	RDS	220	NAAT	Urine	Ng/Ct	27.3
		Tanga					Rectal swab		
Ferré 2019 [59]	Togo	Lomé	Cross-sectional	RDS	207	NAAT	Rectal swab	Ng	11.6
		Kpalimé							
		Atakpamé						Ct	9.7
		Tsévié							
Kim 2016 [55]	Uganda	Kampala	Biobehavioural	RDS	295	NAAT	Urine	Ng	1.4
							Rectal swab	Ct	1.0
Laurent 2021 [63]	Burkina Faso	Ouagadougou	Prospective cohort	Not reported	598	NAAT	Urine	Ng	12.6
	Côte d'Ivoire	Abidjan					Rectal swab		
	Mali	Bamako					Pharyngeal swab	Ct	19.3
	Togo	Lomé							
Ngetsa 2020 [62]	Kenya	Coastal Kenya	Not reported	Not reported	104	NAAT	Rectal swab	Ng	9.6
						Culture		Ct	13.5
Mehta 2021 [64]	Kenya	Kisumu	Prospective cohort	Not reported	158	NAAT	Urine	Ng	1.9
							Rectal swab	Ct	7.7
De Baetselier 2020 [60]	Burkina Faso	Ouagadougou	Not reported	Not reported	497	NAAT	NR	Ng	11.5
	Togo	Lomé							
	Mali	Bamako						Ct	14.5
	Côte d’Ivoire	Abidjan							
Vuylsteke 2012 [51]	Côte d’Ivoire	Abidjan	Cross-sectional	Not reported	94	NAAT	Urine	Ng	12.8
							Rectal swab	Ct	3.2
Wade 2005 [49]	Senegal	Dakar	Epidemiological	Snowball	442	NAAT Serology	Urine Blood	Ng Ct	5.4 4.1
		Thiés							
		Mbour							
		Kaolack							
		Saint-Louis							
Jones 2020 [61]	South Africa	Cape Town	Prospective cohort	Not reported	292	NAAT	Urine	Ng	2.3
		Port Elizabeth					Rectal swab	Ct	10.8
Sanders 2014 [53]	Kenya	Coastal Kenya	Cohort	Not reported	244	NAAT	Urine	Ng	1.6
							Rectal swab	Ct	6.1
Sanders 2010 [50]	Kenya	Coastal Kenya	Cohort	Not reported	43	NAAT	Urine	Ng	2.0
							Rectal swab	Ct	12.0
Mwaniki 2023 [66]	Kenya	Nairobi	Cross-sectional	RDS	242	NAAT	Urine	Ng	14.9
							Rectal swab	Ct	58.7
							Pharyngeal swab		
Le Roux 2023 [65]	South Africa	Tshwane	Not reported	Snowball	199	NAAT	Urethral swab	Ng	17.1
							Rectal swab	Ct	18.1
							Pharyngeal swab		
Mashingaidze 2023 [34]	South Africa	Gauteng	Clinical trial	Not reported	173	NAAT	Urine	Ng Ct	8.1 26.0
		Western Cape							
		KwaZulu-Natal					Rectal swab		
		North West							
		Eastern Cape							
Malefo 2023 [38]	South Africa	Tshwane North	Cross-sectional	RDS	200	Serology	Urethral swab	Ng	9.0
							Rectal swab	Ct	20.0
							Pharyngeal swab		

Abbreviations: *NAAT* nucleic acid amplification tests, *RDS* respondent-driven sampling, *Ng* Neisseria gonorrhoeae, *Ct* Chlamydia trachomatis

Of the studies selected, six were cross-sectional, four studies had a prospective cohort study design, and one was an epidemiological study (Table 1). The respondent-driven sampling (RDS) technique was used by eight studies to reach the MSM populations [52, 54–57, 59, 66]. Three studies [49, 58, 65] used snowball sampling as a recruitment technique, whereas nine studies did not report the sampling method used [34, 50, 51, 53, 60–64]. The majority of the studies (16) employed molecular tests for the diagnosis of Ng and Ct infections [34, 50–57, 59–61, 63–66], whereas one study used serology (rapid plasma reagent test) to test for infections [38]. The remaining studies combined molecular tests with either serology [49] or microscopy/culture tests [58, 62]. Over half the studies (11/20; 55.0%) reported using both urine and rectal swab specimens in testing for Ng and Ct (Table 1).

### Heterogeneity and publication *bias*

The included studies (20) were assessed for heterogeneity and publication bias. There was high heterogeneity for this finding; *I*^2^ = 98% (*Q* = 885.25; *p* = 0.001). The Doi plot for publication bias with an LFK ratio was generated to assess the risk of bias. There was evidence of minor asymmetry with an LFK index of ± 1.20 (see Additional file 2: Figure S1).

### Sensitivity analysis

A sensitivity analysis of the twenty studies was conducted to evaluate the effect each study had on the pooled prevalence data (Table 2). The pooled prevalence, shown as a fraction in the table (second column), did not alter significantly when the particular study in the first column was removed from the meta-analysis. The only study that was remarkable in that it reduced the pooled prevalence by approximately 3% was the removal of De Baetselier et al.’s study [60]. Furthermore, this sensitivity analysis did not impact the heterogeneity. None of the studies, when they were removed from the analysis, increased the prevalence by any significant degree.

**Table 2:** Sensitivity analysis

Excluded study	Pooled prevalence	LCI 95%	HCI 95%	Cochrane *Q*	*P*	*I*^2^	*I*^2^ LCI 95%	*I*^2^ HCI 95%
Quilter et al. 2018 [58]	0.279	0.186	0.384	877.3	0.0	97.9	97.5	98.3
Rebe et al. 2015 [54]	0.274	0.184	0.374	885.0	0.0	98.0	97.5	98.4
Ramadhani et al. 2016 [56]	0.282	0.192	0.382	816.8	0.0	97.8	97.3	98.2
Crowell et al. 2018 [57]	0.278	0.187	0.379	861.3	0.0	97.9	97.4	98.3
Ross et al. 2014 [52]	0.272	0.183	0.371	884.6	0.0	98.0	97.5	98.4
Ferré et al. 2019 [59]	0.273	0.185	0.371	883.7	0.0	98.0	97.5	98.4
Kim et al. 2016 [55]	0.283	0.197	0.378	778.4	0.0	97.7	97.1	98.1
Laurent et al. 2021 [63]	0.276	0.183	0.380	883.9	0.0	98.0	97.5	98.4
Ngetsa et al. 2020 [62]	0.275	0.187	0.374	884.5	0.0	98.0	97.5	98.4
Mehta et al. 2021 [64]	0.279	0.190	0.378	871.1	0.0	97.9	97.4	98.3
De Baetselier et al. 2020 [60]	0.245	0.167	0.334	757.3	0.0	97.6	97.0	98.1
Vuylsteke et al. 2012 [51]	0.270	0.182	0.368	883.5	0.0	98.0	97.5	98.4
Wade et al. 2005 [49]	0.277	0.190	0.374	801.7	0.0	97.8	97.2	98.2
Jones et al. 2020 [61]	0.264	0.176	0.363	862.0	0.0	97.9	97.4	98.3
Sanders et al. 2014 [53]	0.284	0.194	0.384	860.1	0.0	97.9	97.4	98.3
Sanders et al. 2010 [50]	0.273	0.185	0.371	885.2	0.0	98.0	97.5	98.4
Mwaniki et al. 2023 [66]	0.258	0.184	0.340	626.3	0.0	97.1	96.4	97.7
Le Roux et al. 2023 [65]	0.269	0.181	0.366	867.1	0.0	97.9	97.4	98.3
Mashingaidze et al. 2023 [34]	0.268	0.181	0.365	862.6	0.0	97.9	97.4	98.3
Malefo et al. 2023 [38]	0.261	0.181	0.350	736.2	0.0	97.6	96.9	98.0

### Pooled prevalence of *N. gonorrhea* and *C. trachomatis* among MSM

**Fig. 2:**
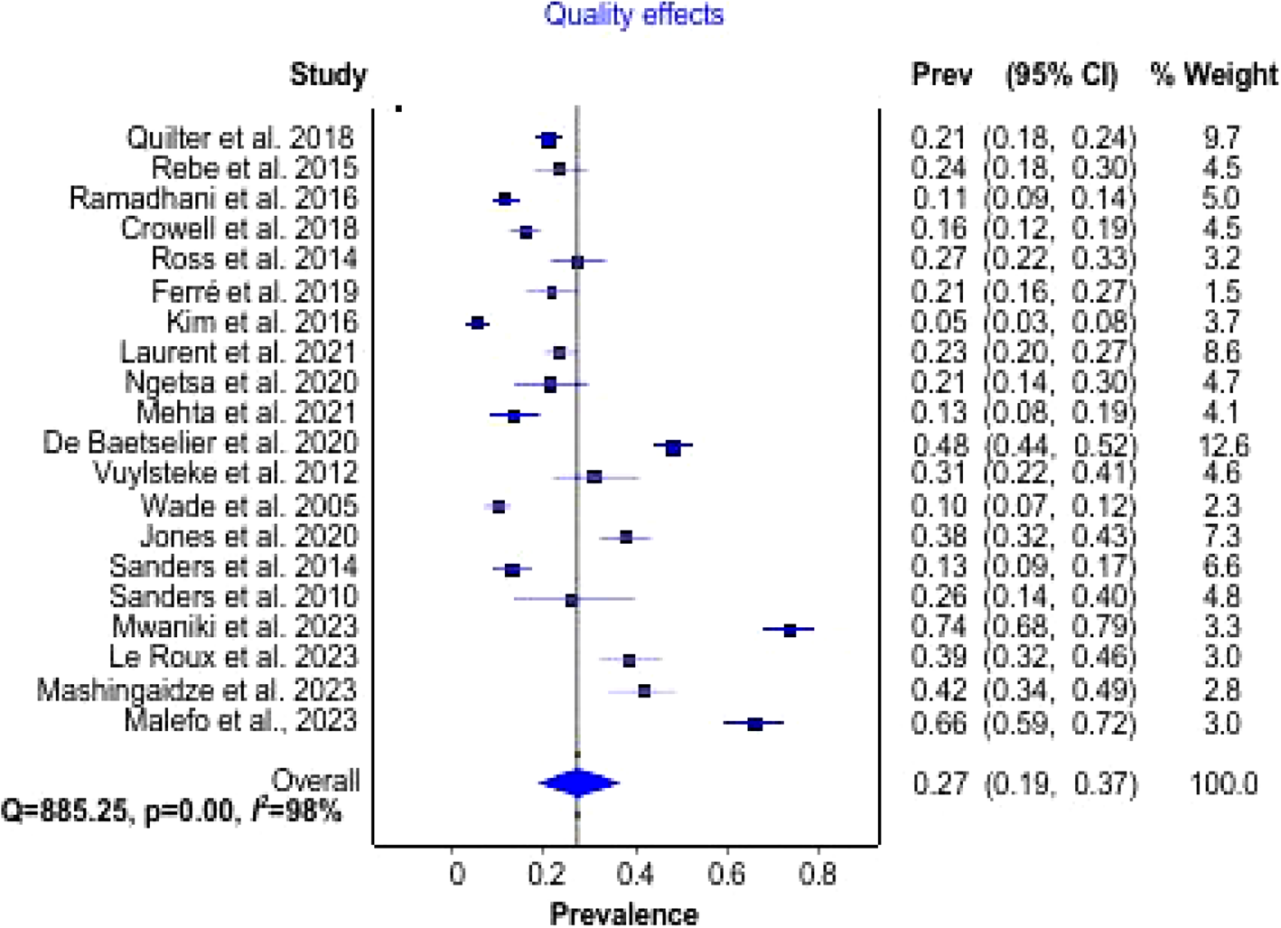
Forest plot of the pooled prevalence of *N. gonorrhea* and *C. trachomatis* among MSM in SSA

The prevalence estimates of Ng and Ct among MSM are presented in a forest plot (Fig. 2). The overall prevalence of the meta-analysis of 20 studies using the quality effects model [46] revealed that the pooled prevalence of Ng and *C*t among MSM in SSA was 27% (95% CI; 19–37%). The *I*^2^ value (98%) which suggests significant heterogeneity is reflective of differences in the sampled populations in SSA. To investigate the heterogeneity, subgroup analyses were conducted using country and study design.

**Fig. 3:**
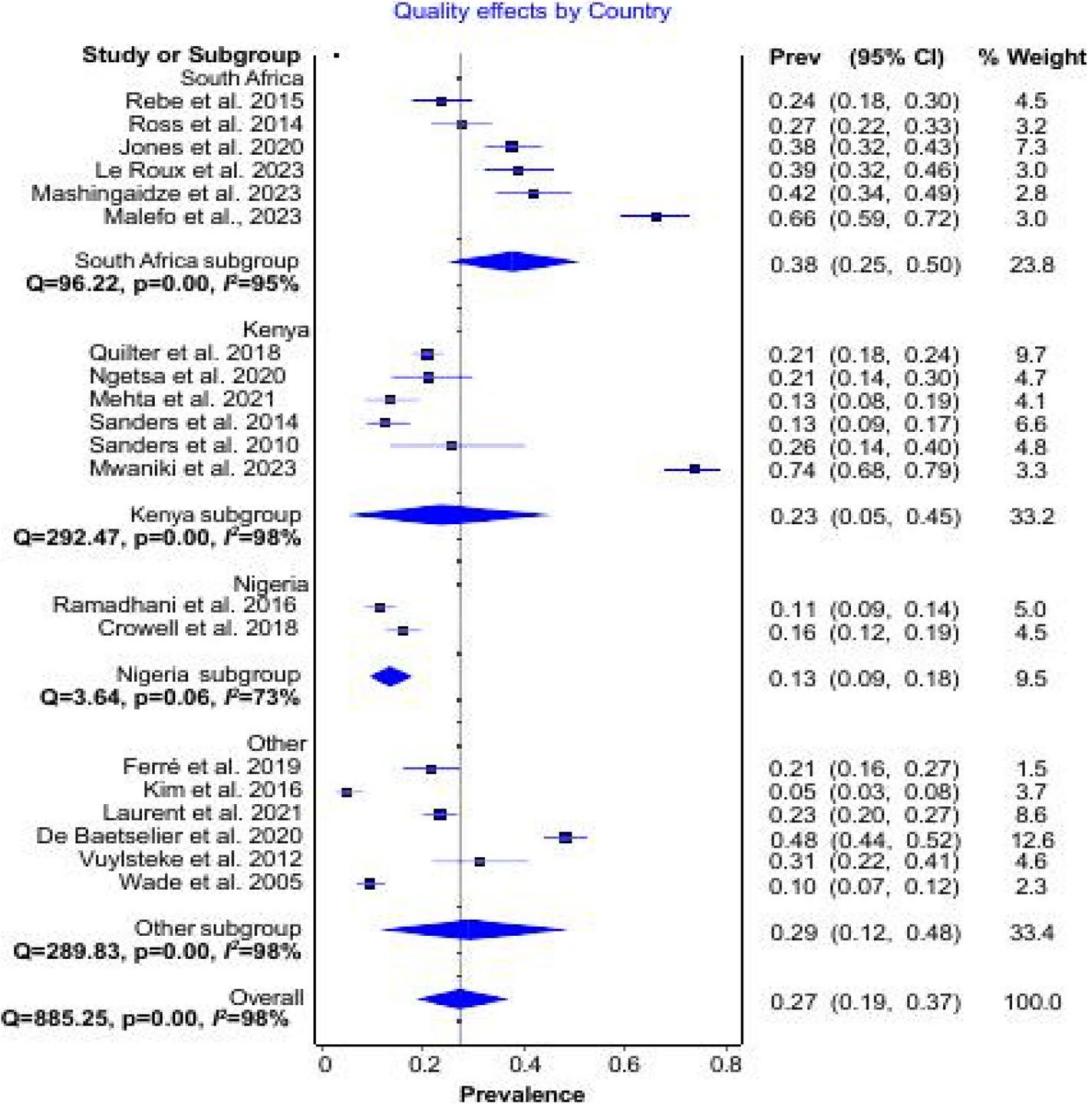
Subgroup analysis of *N. gonorrhea* and *C. trachomatis* prevalence by country of study

Subgroup analysis by country (Fig. 3) indicated that South Africa (*n* = 6) has a prevalence of 38% (95% CI; 25–50%). Kenya (*n* = 6) was 23% (95% CI; 5–45%), and Nigeria (*n* = 2) was 13% (95% CI; 9–18%). Other countries (*n* = 6) were grouped together due to fewer studies conducted in these countries (Burkina Faso, Côte d’Ivoire, Mali, Senegal, Tanzania, Togo, and Uganda), and the prevalence was estimated at 29% (95% CI; 12–48%).

**Fig. 4:**
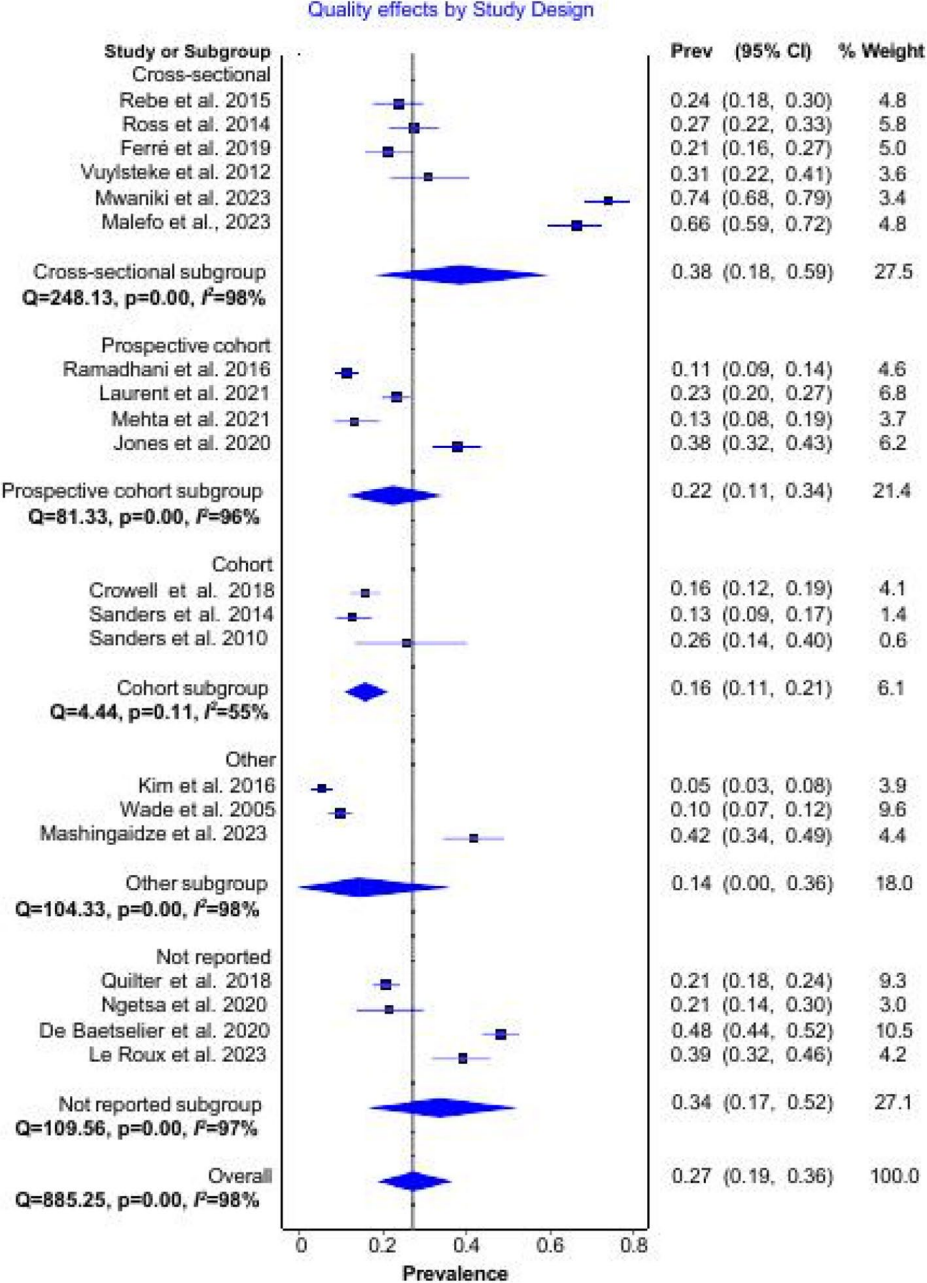
Subgroup analysis of *N. gonorrhea* and *C.* *trachomatis* prevalence by the study design

In terms of study design, the included studies were separated into five (cross-sectional, prospective cohort, cohort, other, and not reported) groups. Subgroup analysis by study design (Fig. 4) showed heterogeneity in all groups but a reduction in the cohort-type studies (*I*^*2*^ = 55%). When we considered prevalence based on study design, we found that cross-sectional type studies showed a higher pooled prevalence of 38% (95% CI; 18–59%) compared to the overall pooled prevalence estimate of 27% (95% CI; 19–37%). The prevalence derived from cohort-type studies was 16% (95% CI; 11–21%), revealing a lower pooled result.

## Discussion

Men who have sex with men (MSM) infected with gonorrhea and chlamydia are at greater risk of acquiring HIV [67]. To reduce this risk, screening of the MSM population and treatment for asymptomatic chlamydia and gonorrhea must be prioritized [68].

We conducted a systematic review and meta-analysis of studies on gonorrhea and chlamydia among MSM in SSA countries. To the best of our knowledge, this is the first attempt to estimate the pooled prevalence of Nt/Ct among MSM in SSA. Overall, most of the studies employed molecular tests for the diagnosis of *N. gonorrhea* and *C. trachomatis* infections in MSM [34, 50–57, 59–61, 63–66]. The nucleic acid amplification tests (NAATs) are the most sensitive techniques for the detection of Ng [69] and Ct [70]. Most of these tests are based on polymerase chain reaction (PCR) and have high specificity [70]. For Ng NAATs, sensitivity and specificity are generally > 95% and > 99% in male first-catch urine [71, 72]. Although NAATs remain the most reliable assays for the detection of pathogens, their use in low-income countries is greatly limited due to relatively high costs [69]. A number of studies have shown that the results of different NAATs were highly concordant [73, 74].

Two fifths (8/20) of the studies used RDS as a sampling technique to reach MSM. The data from this review suggest that RDS is the most commonly used method for sampling MSM. This scientific method is used to recruit populations that are difficult to access [35]. It is characterized by the sample to be studied being created by the MSM themselves through chain referrals [75–77]. Although the RDS is a variant of “snowball sampling,” it has been shown to produce unbiased estimates under certain conditions [78–80].

The prevalence of Ng varied from 1.4% to 47.2% [55, 58]. The lowest prevalence rate of Ng was reported in Uganda in 2016 [55], while the highest prevalence was reported in 2018 among MSM in Kenya [58]. For Ct, the prevalence ranged from 1.0% in 2016 (Uganda) [55] to 58.7% in 2023 (Kenya) [66]. *Chlamydia trachomatis* is the most common bacterial STI with a global estimate of 105.7 million new infections occurring annually [81]. A systematic review by Dewart et al. on the prevalence of rectal Ct and Ng in MSM and women revealed that although Ct and Ng were the most prevalent STIs among MSM, chlamydia was more prevalent when compared with gonorrhea [9]. Studies among community-recruited MSM have also shown that infection with gonorrhea is far less common than chlamydia infection [82, 83]. Contrasting trends, however, have been reported in other areas. A study conducted among MSM in the cities of Agadir and Fes in 2020 reported an overall prevalence of 11.3% and 13.3% for Ct and Ng, respectively [84]. The predominant asymptomatic presentation of Ct, which allows for a longer duration of infection and subsequent transmission, may be responsible for the high prevalence of the infection worldwide [68, 85].

Meta-analysis can obtain a large sample size while providing a strong and reliable prevalence estimate [86] for Ng/Ct. This study estimated the overall prevalence of Ng/Ct among MSM in SSA by reviewing the findings of 20 studies. The overall result of the meta-analysis gave a pooled prevalence of 27% (95% CI, 19–39%), with an *I*^*2*^ of 98% which indicates high heterogeneity among the studies. This pooled prevalence is comparable with reports from Tanzania MSM with a prevalence rate of 27.3% [52]. A meta-analysis conducted by WHO reported that the global prevalence of Ng and Ct among men was 0.6% and 2.7%, respectively [15]. Also, in 2019, Rowley et al. performed a systematic review to estimate the global prevalence of Ng/Ct in men. The prevalence estimates were Ng 0.7% and Ct 2.7% [12]. Findings from our review suggest that the burden of Ng and Ct among MSM in SSA is higher when compared with other regions.

We assessed publication bias by using the Doi plot and LFK ratio which showed minor asymmetry, suggesting there was limited publication bias [48]. However, due to the paucity of relevant studies in the region, and that the selected studies reported on either prevalence if there were cross-sectional or baseline prevalence in cohort studies, there would be a reduction in the impact of publication bias as there was little dependency on positive statistical outcomes. Furthermore, we used a quality effects model [47], as opposed to a random effects model, where selected studies were weighted based on the quality as determined by the Hoy tool [41]. This allowed for a more rational determination of the pooled prevalence given the limited number of participants in the individual studies. To find the source of, and reduce the heterogeneity, we performed subgroup analyses by country as well as by study design.

When refining the analysis by country, South Africa had a high Ng/Ct prevalence rate (38%), followed by Kenya (23%). Based on our analysis, Ng/Ct prevalence was estimated to be the lowest in Nigeria (13%). Sexually transmitted infections have been reported to be highly prevalent among MSM in South Africa [61, 87]. In South Africa, the prevalence of reported asymptomatic STIs varies wildly, ranging from 19 to 90% [54, 87, 88]. Among South African MSM, 91% of diagnosed rectal Ng and/or Ct infections were clinically asymptomatic [61]. A study conducted in Tshwane North, South Africa, among MSM, reported 66% STI prevalence [38], higher than in other studies conducted in Tanzania and Kenya. The results of the study conducted in Tanzania, among MSM, reported gonorrhea, chlamydia, and syphilis rates of 21% [52]. Similarly, a study on MSM from Kenya found that 26% tested positive for Ct, Ng, or both [50]. Also, few studies on MSM in South Africa have reported a prevalence of 10%–24% for Ct and 3%–55% for Ng at any anatomic site regardless of the presence of symptoms [54, 61, 87]. Although South Africa is the sole country in Africa where MSM rights are protected by the constitution [89], making it much easier to access this key population, it still remains that Ng/Ct is prevalent and the burden among South African MSM is high.

Kenya has been at the forefront of recognizing the vulnerabilities of MSM who feared legal authorities and had virtually no access to health services [90]. However, despite negative public debates and legal challenges [91], the Kenyan Ministry of Health/National AIDS and STI control programs have recognized that MSM is a key population in need of urgent attention and have demonstrated their willingness to work with them [92]. This is reflective in the number of research studies emanating from the country, some of which have been included in our study. In this study, the overall prevalence of Ng/Ct in Kenya was 23%. The observed prevalence is comparable to that observed among MSM in coastal Kenya (26%) [50]. By contrast, the overall Ng/Ct prevalence among MSM in our study was higher than that in the general population in Kenya, as reported in a study that found the prevalence of chlamydia and gonorrhea were 16.8% and 7.1%, respectively [93]. This observation may be due to factors such as transactional sexual intercourse, unprotected anal intercourse, and being HIV-positive, which have been found to be associated with Ng/Ct [58, 94–96].

Our study showed that the overall prevalence of Ng/Ct infections in Nigeria was 13%. The prevalence is higher in our study when compared with the 4.2% prevalence of both infections among MSM in Lagos [97]. Similarly, universal screening programs deployed in Tanzania, Botswana, and Kenya have reported high STI prevalence rates of between 12 and 20% in a relatively young MSM population [52, 98, 99]. This finding indicates that these infections are prevalent in the country. However, the observed prevalence should be treated with caution as it may not reflect the true prevalence of infections due to the criminalization of homosexuality [36]. In many of the countries across SSA, sexual intercourse between people of the same sex is criminalized, including in Nigeria [100]. In addition, MSM face significant social stigma and internalized homophobia that may pose as barriers to seeking healthcare services including screening for HIV and other STIs [36, 101]. Unfortunately, there remains a paucity of studies to ascertain the true prevalence and therefore we recommend further study to evaluate the burden of STIs in Nigerian MSM. To achieve the WHO’s goal of ending STI epidemics as major public health concerns [13], countries need to know their STI burden to understand where and among which population groups new infections are occurring [102]. Only then can deliberate actions be effectively taken.

A subgroup analysis by study design was performed to if there was variance in prevalence based on the type of study and if it could explain the heterogeneity found. Our analysis showed that the pooled prevalence changed based on the study designs. For cross-sectional type studies, the pooled prevalence was higher compared to the overall pooled prevalence. Cohort types studies, on the other hand, revealed a lower pooled prevalence. This finding indicates that the study design is responsible for some variation in our pooled prevalence, suggesting that much-needed further research employs uniformity in study design to improve confidence in the prevalence estimates. In addition, the subgroup analysis revealed that study design was responsible for some of the heterogeneity observed, with decreased heterogeneity found in cohort-type studies. However, this should be treated with caution as we also found that some studies were of poor quality particularly and suffered from small sample sizes. Therefore, while there needs to be an increase in the number of studies spanning the entire region, improving the sample size may improve confidence in the prevalence estimates. This may require a multifaceted approach that involves awareness and education campaigns that are designed not only for prevention targets but also aimed at reducing stigma, in addition to standard test and treatment protocols. This may have the advantage of improving sampling, especially in countries where MSM is treated with abhorrence.

This is the first study to systematically review the available literature on the prevalence of Ng/Ct among MSM in SSA. However, our study had limitations. First, limited data reporting on the prevalence of Ng/Ct were available, making it difficult to ascertain the true prevalence and associated risk factors in SSA. Second, our search on the databases was limited to only studies reported in English which may have resulted in the exclusion of studies published in languages other than English.

## Conclusion

This systematic review and meta-analysis revealed a pooled prevalence of Ng/Ct in the MSM population to be 27% (95% CI, 19–37%). Efforts were made to find and analyze the available data to provide a unique perspective on this issue, with the aim of informing policymakers on the need to prioritize MSM healthcare needs. Given the high prevalence observed in the MSM population, targeted interventions such as male circumcision, partner testing, and extensive health education are needed. More importantly, it is crucial to ensure the use of better diagnostic methods that will lead to accurate STI testing and appropriate treatment as well as minimize syndromic management approach to treatment. Furthermore, future studies need to consider a uniform method to establish prevalence or at least improve scientific rigor by reporting the study design used.

## Supplementary Information

Supplementary file 1

Supplementary file 2

## Data Availability

All data generated or analyzed during this investigation is included in the published systematic review article and will be available upon request.

## Abbreviations

MSM: Men who have sex with men
STIs: Sexually transmitted infections
Ng: *Neisseria gonorrhoeae*
Ct: *Chlamydia trachomatis*
SSA: Sub-Saharan Africa
HIV: Human immunodeficiency virus
AIDS: Acute immunodeficiency syndrome
PID: Pelvic inflammatory disease
NAAT: Nucleic acid amplification tests
PCR: Polymerase chain reaction
FCSW: Female commercial sex workers
TGW: Transgender women
RDS: Respondent-driven sampling
DOAJ: Directory of Open Access Journals
PRISMA: Preferred Reporting Items for Systematic Reviews and Meta-analyses
MeSH: Medical Subject Headings
GRADE: Grading of Recommendations, Assessment, Development and Evaluation
CI: Confidence interval
