# Supplementary material for: Prevalence of gonococcal and chlamydial infections among men who have sex with men in sub-Saharan Africa: a systematic review and meta-analysis
Source: Syst Rev. 2024 Nov 16;13:282. doi: 10.1186/s13643-024-02704-z (PMC11568532; doi:10.1186/s13643-024-02704-z)
Supplement: Supplementary file 1 [file 13643_2024_2704_MOESM1_ESM.docx]

**Supplementary material**

**Table S1** Risk of bias assessment of included studies using the Hoy tool [41]

| **Study ID** | **Representation** | **Sampling frame** | **Sample selection** | **Non-response**  **bias** | **Data collection** | **Case Definition** | **Reliability and validity of study tool** | **Method of data collection** | **Numerator and denominator** | **Summary Assessment** |
| --- | --- | --- | --- | --- | --- | --- | --- | --- | --- | --- |
| Quilter (2018)^58^ | High risk | Not clear | Low risk | Not clear | Low risk | Low risk | Low risk | Low risk | Low risk | Medium risk |
| Rebe (2015)^54^ | High risk | Low risk | Low risk | Not clear | Low risk | Low risk | High risk | Low risk | Low risk | Medium risk |
| Ramadhani (2016)^56^ | High risk | Low risk | Low risk | Not clear | Low risk | Low risk | Low risk | Low risk | Low risk | Low risk |
| Crowell (2018)^57^ | High risk | Low risk | Low risk | Not clear | Low risk | Low risk | Not clear | Low risk | Low risk | Medium risk |
| Ross (2014)^52^ | High risk | Not clear | Low risk | Low risk | Low risk | Low risk | Low risk | Low risk | Low risk | Low risk |
| Ferré (2019)^59^ | Low risk | Low risk | Low risk | Not clear | Low risk | Low risk | Low risk | Low risk | Low risk | Low risk |
| Kim (2016)^55^ | High risk | Low risk | Low risk | Not clear | Low risk | Low risk | Low risk | Low risk | Low risk | Low risk |
| Laurent (2021)^63^ | Not clear | Low risk | Not clear | Not clear | Low risk | Low risk | Low risk | Low risk | Low risk | Medium risk |
| Ngetsa (2020)^62^ | Not clear | Low risk | Not clear | Not clear | Low risk | Low risk | High risk | Low risk | Low risk | Medium risk |
| Mehta (2021)^64^ | High risk | Low risk | Not clear | Not clear | Low risk | Low risk | Low risk | Low risk | Low risk | Medium risk |
| De Baetselier (2020)^60^ | Not clear | Not clear | Not clear | Not clear | Low risk | Low risk | Not clear | Low risk | Low risk | High risk |
| Vuylsteke (2012)^51^ | High risk | Low risk | Not clear | Not clear | Low risk | High risk | Low risk | Low risk | Low risk | Medium risk |
| Wade (2005)^49^ | Low risk | Low risk | Low risk | Not clear | Low risk | Low risk | Low risk | Low risk | Low risk | Low risk |
| Jones (2020)^61^ | High risk | High risk | Not clear | Not clear | Low risk | Low risk | Low risk | Low risk | Low risk | Medium risk |
| Sanders (2014)^53^ | Not clear | Low risk | High risk | Not clear | Low risk | Low risk | Not clear | Low risk | Low risk | Medium risk |
| Sanders (2010)^50^ | Not clear | Not clear | High risk | Not clear | Low risk | Low risk | Not clear | Low risk | Low risk | High risk |
| Mwaniki (2023)^66^ | High risk | Low risk | Low risk | Not clear | Low risk | Low risk | Low risk | Low risk | Low risk | Low risk |
| Le Roux (2023)^65^ | Not clear | Low risk | Low risk | Not clear | Low risk | Low risk | Low risk | Low risk | Low risk | Low risk |
| Mashingaidze (2023)^34^ | Low risk | Low risk | Not clear | Not clear | Low risk | Low risk | Low risk | Low risk | Low risk | Low risk |
| Malefo (2023)^38^ | High risk | Low risk | Low risk | Not clear | Low risk | Low risk | Low risk | Low risk | Low risk | Low risk |

**Risk of bias assessment tool: Yes (low risk); No (high risk)**

1. Representation: Was the study population a close representation of the national population?
2. Sampling: Was the sampling frame a true or close representation of the target population?
3. Sample selection: was some form of sampling strategy used to select the sample?
4. Non-response bias: Was the likelihood of non-response bias minimal?
5. Data collection: Were data collected directly from the subjects?
6. Case definition: Was an acceptable case definition used in the study?
7. Reliability and validity of study tool: Was the study instrument that measured the parameter of interest show to have reliability and validity?
8. Data collection: Was the same mode of data collection used for all subjects?
9. Numerators and denominators: Were the numerator(s) and denominator(s) for the parameter of interest appropriate?

**The overall risk of bias was scored according to the number of high risk of bias per study: low (≤2), moderate (3–4), and high (≥5).**
