# Supplementary material for: Prevalence of gonococcal and chlamydial infections among men who have sex with men in sub-Saharan Africa: a systematic review and meta-analysis
Source: Syst Rev. 2024 Nov 16;13:282. doi: 10.1186/s13643-024-02704-z (PMC11568532; doi:10.1186/s13643-024-02704-z)
Supplement: Supplementary file 2 [file 13643_2024_2704_MOESM2_ESM.docx]

**Figure S1.** Doi plot analysis and LFK index of publication bias and asymmetry of overall pooled prevalence
